# Do cancer patients with dementia receive less aggressive treatment in end-of-life care? A nationwide population-based cohort study

**DOI:** 10.18632/oncotarget.18867

**Published:** 2017-06-29

**Authors:** Huei-Kai Huang, Jyh-Gang Hsieh, Chia-Jung Hsieh, Ying-Wei Wang

**Affiliations:** ^1^ Department of Family Medicine, Buddhist Tzu Chi General Hospital, Hualien, Taiwan; ^2^ School of Medicine, Tzu Chi University, Hualien, Taiwan; ^3^ Department of Public Health, Tzu Chi University, Hualien, Taiwan

**Keywords:** cancer, dementia, palliative care, terminal care, end-of-life care

## Abstract

Dementia is a progressive, incurable disease that can deprive patients of the ability to make decisions. This study determines whether dementia influences the medical care that a cancer patient receives at the end of life. We conducted a nationwide population-based cohort study on patients aged ≥20 with newly diagnosed cancer during 2000–2012. After matching to reduce confounders, there were 7,111 patients with and 28,444 without dementia. The adjusted odd ratios (OR) for medical interventions, including intensive care, palliative care, invasive procedures, and advanced diagnostic testing, were calculated for the final month and three months of life by a multiple logistic regression model. In the final month before death, the dementia cohort had longer hospital stays (17.7 vs. 17.1 days), more intensive care unit stays (OR = 1.32), and less palliative care (OR = 0.80) than the non-dementia cohort and were more likely to receive invasive procedures, including cardiopulmonary resuscitation (OR = 1.32), endotracheal intubation (OR = 1.27), mechanical ventilation (OR = 1.45), urinary catheterization (OR = 1.24), and feeding tube (OR = 1.88), but less likely to undergo chemotherapy (OR = 0.60) and diagnostic procedures such as computed tomography, magnetic resonance imaging, and sonography (OR = 0.87) or bone scan (OR = 0.69). The analysis examining the three months before death had similar results. In summary, patients with cancer and dementia are more likely to receive intensive care and invasive procedures but less likely to undergo advanced diagnostic testing, chemotherapy, or hospice care than those with cancer but without dementia.

## INTRODUCTION

Despite the possibility of early detection and substantial improvements in treatment and survival, cancer remains the leading cause of death in most developed countries [1]. In Taiwan, cancer accounted for 28.6% of all deaths in 2015 [2]. Even though greater importance is being attached to the quality of medical care provided to terminal cancer patients [3] and a growing body of evidence indicates that treatment intensity is not positively correlated with health outcome or satisfaction [4–6], the aggressiveness of end-of-life care in cancer patients is increasing [1, 7]. Admission to the intensive care unit and receiving chemotherapy in the end-of-life were considered as aggressive treatments. On the other hand, receiving palliative care program was considered as a less aggressive treatment [8]. Aggressive treatment administered at the end-of-life may confer disproportionately small benefits. Moreover, aggressive treatment can pose society-wide economic burdens [9].

Dementia is a progressive, incurable disease that can deprive patients of the ability to make decisions for end-of-life care [10]. If the wishes of patients with dementia are unknown, they may be subjected to more invasive intervention at the end of life compared to those without dementia [11]. At present, few studies have investigated the influence of dementia on aggressive end-of-life care, and the conclusions drawn by these studies have been inconsistent. For example, Afzal et al. found that invasive interventions administered to patients with dementia did not differ from those administered to patients without dementia, although the former received hospice care less frequently [12]. However, other studies have reported that patients with dementia receive fewer invasive interventions than other patients [6, 13, 14].

Although a significant overlap of cancer and dementia can be expected because age is a major risk factor for both conditions [15, 16], little research has been focused on the caring of those with both dementia and terminal cancer [6]. However, these issues are important for the specific population because individuals with dementia may have impairment in communicating and understanding that may increase the difficulty in diagnosing or treating cancer and discussing the issues related to death and dying. Patients’ family members have to make surrogate decisions for them when they lack the capacity to do so, and this can be distressing for both groups.

To address these research gaps, we used nationwide, population-based data from Taiwan to compare medical care administered to patients with terminal cancer with and without dementia during the final months prior to death.

## RESULTS

### Patient characteristics

**Figure 1:**
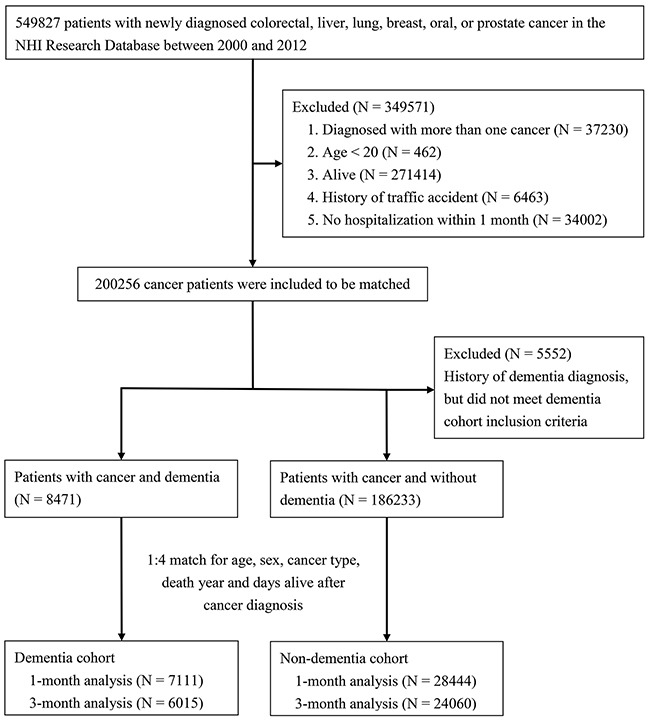
Flow diagram for selection of study subjects

We identified 549,827 patients with newly diagnosed colorectal, liver, lung, breast, oral, or prostate cancer from the nationwide cohort between 2000 and 2012. After excluding patients not meeting study criteria (n = 349,571), we matched 7,111 patients with both cancer and dementia to 28,444 patients with cancer but without dementia (Figure 1). Variables, including age, gender, type of primary cancer, and length of time between cancer diagnosis and death, were balanced between the two groups of patients. Insurance premium (as a proxy of income) and Charlson comorbidity index varied slightly between the groups (Table 1).

**Table 1:** Demographic characteristics and diagnoses of study patients

	Dementia (N = 7111)		Non-dementia (N = 28444)	
	N	%	N	%
Cancer type				
Colorectal cancer	1942	27.3	7768	27.3
Liver cancer	1602	22.5	6408	22.5
Lung cancer	1948	27.4	7792	27.4
Breast cancer	300	4.2	1200	4.2
Oral cancer	301	4.2	1204	4.2
Prostate cancer	1018	14.3	4072	14.3
Age at death (years)				
<70	590	8.3	2360	8.3
70-74	782	11.0	3128	11.0
75-79	1507	21.2	6028	21.2
80-84	2003	28.2	8012	28.2
≥85	2229	31.4	8916	31.4
Gender				
Male	4521	63.6	18084	63.6
Female	2590	36.4	10360	36.4
Year of death				
2000–2003	349	4.9	1396	4.9
2004–2007	1865	26.2	7460	26.2
2008–2012	4897	68.9	19588	68.9
Cancer diagnosis to death* (years)	2.19	2.53	2.02	2.33
Insurance premium (NTD)				
Financially dependent	2096	29.5	8215	28.9
1–15840	1557	21.9	5496	19.3
15841–25000	2937	41.3	12636	44.4
25001	521	7.3	2095	7.4
Charlson comorbidity index				
0–3	1759	24.7	11822	41.6
4–6	3545	49.9	10321	36.3
7–9	1189	16.7	4368	15.4
≥10	618	8.7	1933	6.8

*Mean and SD

NTD, New Taiwan dollars

### Medical care utilization

The 1-month analysis (i.e., analysis of care given within 1 month prior to death) revealed that patients with both cancer and dementia had longer hospital stays than those without dementia (17.7 vs. 17.1 days, p < 0.0001). They also had a higher likelihood of intensive care unit (ICU) admission (OR = 1.32, 95% CI 1.25–1.39) and a lower likelihood of receiving palliative care (OR = 0.80, 95% CI 0.74–0.86), including hospice ward care (OR = 0.85, 95% CI 0.79–0.92) and palliative consultation service (OR = 0.61, 95% CI 0.51–0.72) (Table 2). However, the two groups had no significant difference in terms of the hospice home care they received (OR = 1.05, 95% CI 0.94–1.17). The 3-month analysis (i.e., the analysis of care given within the 3 months prior to death) showed results similar to the 1-month analysis (Table 2).

**Table 2:** Comparison of utilization of medical care and palliative care service between cancer patients with and without dementia 1 and 3 months prior to death

	1 month prior to death					3 months prior to death				
	Dementia (N = 7111)		Non-dementia (N = 28444)			Dementia (N = 6015)		Non-dementia (N = 24060)		
	N	%	N	%	Adjusted OR (95% CI)†	N	%	N	%	Adjusted OR (95% CI)†
Inpatient day‡ (days, Mean ± SD)	17.7 ± 10.3		17.1 ± 10.1		p < 0.0001***	33.8 ± 26.4		30.2 ± 24.0		p < 0.0001***
Intensive care	2768	38.9	9192	32.3	1.32 (1.25–1.39)***	2668	44.4	8628	35.9	1.39 (1.31–1.48)***
Palliative care	1142	16.1	5529	19.4	0.80 (0.74–0.86)***	966	16.1	4807	20.0	0.77 (0.71–0.83)***
Hospice ward care	1014	14.3	4683	16.5	0.85 (0.79–0.92)***	857	14.3	4002	16.6	0.84 (0.78–0.91)***
Palliative consultation service	180	2.5	1131	4.0	0.61 (0.51–0.72)***	172	2.9	1122	4.7	0.58 (0.49–0.69)***
Hospice home care	463	6.5	1776	6.2	1.05 (0.94–1.17)	473	7.9	1823	7.6	1.05 (0.95–1.17)

†Multiple logistic regression with adjusted for age, sex, primary malignancy, Charlson comorbidity index, year of death, and insurance premium.

‡P values for differences in means determined by independent t test

*p < 0.05, **p < 0.01, ***p < 0.0001

### Use of invasive procedures and advanced diagnostic testing

Overall, patients with both cancer and dementia received more invasive procedures but less chemotherapy and advanced diagnostic testing than those without dementia.

In the 1-month analysis, patients in the dementia group had a higher likelihood of receiving invasive procedures, including cardiopulmonary resuscitation (CPR) (OR = 1.32, 95% CI 1.22–1.43), endotracheal intubation (OR = 1.27, 95% CI 1.19–1.34), mechanical ventilation (OR = 1.45, 95% CI 1.37–1.53), urinary catheterization (OR = 1.24, 95% CI 1.18–1.30), or a feeding tube (OR = 1.88, 95% CI 1.76–2.00). However, a smaller proportion of patients with dementia received chemotherapy (OR = 0.60, 95% CI 0.55–0.66). They were also less likely to undergo diagnostic imaging, including computed tomography (CT), magnetic resonance imaging (MRI), or sonography (OR = 0.87, 95% CI 0.82–0.92) and bone scans (OR = 0.69, 95% CI 0.61–0.77). There were no significant differences between the two in terms of panendoscopy, colonoscopy, and positron emission tomography (PET) scans (Table 3).

**Table 3:** Comparison of utilization of chemotherapy, invasive procedure, and advanced diagnostic testing between cancer patients with and without dementia 1 and 3 months prior to death

	1 month prior to death					3 months prior to death				
	Dementia (N = 7111)		No dementia (N = 28444)			Dementia (N = 6015)		No dementia (N = 24060)		
	N	%	N	%	Adjusted OR (95% CI)†	N	%	N	%	Adjusted OR (95% CI)†
Chemotherapy	762	10.7	4648	16.3	0.60 (0.55–0.66)***	1104	18.4	7066	29.4	0.51 (0.47–0.55)***
Invasive procedure										
Cardiopulmonary resuscitation	974	13.7	2982	10.5	1.32 (1.22–1.43)***	879	14.6	2647	11.0	1.35 (1.24–1.47)***
Endotracheal intubation	2044	28.7	6833	24.0	1.27 (1.19–1.34)***	1988	33.1	6423	26.7	1.34 (1.26–1.42)***
Mechanical ventilation	3196	44.9	10317	36.3	1.45 (1.37–1.53)***	2886	48.0	9194	38.2	1.49 (1.40–1.50)***
Urinary catheterization	4558	64.1	16731	58.8	1.24 (1.18–1.30)***	4154	69.1	15116	62.8	1.30 (1.22–1.38)***
Feeding tube	5679	79.9	19192	67.5	1.88 (1.76–2.00)***	4921	81.8	16642	69.2	1.96 (1.82–2.10)***
Advanced diagnostic testing										
CT/MRI/Sonography	4263	60.0	17985	63.2	0.87 (0.82–0.92)***	4696	78.1	19572	81.4	0.78 (0.72–0.83)***
Panendoscopy	924	13.0	3862	13.6	0.93 (0.86–1.00)	1215	20.2	4995	20.8	0.91 (0.85–0.90)**
Colonoscopy	198	2.8	683	2.4	1.15 (0.98–1.35)	242	4.0	934	3.9	1.00 (0.87–1.16)
Bone scan	371	5.2	2148	7.6	0.69 (0.61–0.77)***	608	10.1	3619	15.0	0.62 (0.56–0.68)***
PET scan	20	0.3	105	0.4	0.80 (0.49–1.30)	28	0.5	186	0.8	0.61 (0.40–0.91)*

†Multiple logistic regression with adjusted for age, sex, primary malignancy, Charlson comorbidity index, year of death, and insurance premium.

*p < 0.05, **p < 0.01, ***p < 0.0001

The 3-month analysis yielded identical patterns to those of the 1-month analysis with regard to invasive procedures. Significantly fewer patients in the dementia group had a CT, MRI, sonography, or a bone scan. In the 3-month analysis, they also underwent significantly fewer panendoscopies (OR = 0.91, 95% CI 0.85–0.90) and PET scans (OR = 0.61, 95% CI 0.40–0.91) (Table 3).

## DISCUSSION

This nationwide, population-based, retrospective cohort study of patients with terminal cancer found that those with dementia were more likely to be admitted to the ICU and receive invasive procedures, but less likely to be given chemotherapy, receive palliative care, or undergo advanced diagnostic testing than those without dementia.

We found that patients with both cancer and dementia were less likely to receive chemotherapy; this was consistent with results of a previous research [6]. A systematic literature review found that elderly patients tend to follow doctors’ suggestions when deciding whether to undergo chemotherapy [17]. Moreover, a previous study conducted in Asia observed that performance status and comorbidities are the two main factors that doctors consider when deciding about the administration of chemotherapy [18]. This helps explain why a lower proportion of patients with dementia received chemotherapy than the matched patients without dementia.

Conversely, our findings of a higher likelihood for invasive procedures in patients with dementia are not entirely consistent with those of previous studies [6, 12–14]. There are several possible explanations for the discrepancy.

**1. The relationship between culture and medical decisions**: Previous research has shown that cultural attitudes toward truth-telling, life-extension, and end-of-life decisions vary significantly, particularly between Western and Asian cultures [19–22]. These cultural factors can influence whether patients with dementia undergo more aggressive interventions at the end of life. In countries such as Taiwan, where “family consent for disclosure” and “family autonomy” are prominent, diagnosis and prognosis are often revealed to family members before the patients themselves are told. Therefore, family members may make the medical decisions, even when the patient is completely lucid [23]. A research conducted in Taiwan found that end-of-life care preferences of patients with cancer and their family caregivers may differ more than they do in Western countries. Family members may be more accepting of aggressive care and assertive in requesting it than are patients themselves when it comes to invasive and life-support measures [21]. Another study found that family members tend to want patients with dementia to receive invasive procedure near the end of their lives, but they would refuse such treatment for themselves under similar circumstances [24]. Since families tend to dominate medical decision making even for competent patients, it is especially true for patients with dementia who cannot clearly express their own wishes. Moreover, eastern culture is a high-context culture. In other words, people tend to address issues such as illness less directly and explicitly than Westerners. Therefore, in emergency situations, doctors often ask family members to decide whether invasive procedures such as CPR or endotracheal intubation should be administered. However, this action is not consistent with the habits in Eastern, high-context culture, which can influence the decisions of family members [25]. A previous study in terminally ill Taiwanese cancer patients showed that the patient's awareness of prognosis, patient–family caregiver congruence on the preferred place of death, and the subjective family caregiving burden had a significant impact on the quality of life [26]. This gives further prominence to the importance of the role of family caregiver in Asian cultures such as in Taiwan.

**2. Lack of advance directives**: Advance directives are rarer in Taiwan than in other countries [27, 28]. In traditional Chinese culture, people are less willing to talk about death. Indeed, talking about death with elderly individuals is taboo because it is believed to bring bad fortune. Even with the constant promotion of hospice care in recent years, children are expected to provide their parents with high-intensity treatment unless the parents themselves direct otherwise. If the family members do not insist on aggressive treatment, others may see them as having given up on their parents or lacking in filial piety [29, 30]. Therefore, in the absence of advance directives, family members of patients with dementia can have difficulty accepting hospice care or reducing the number of invasive interventions, even if a doctor suggests that doing so is the best course of action.

**3. Influence of National Health Insurance (NHI)**: The Taiwanese healthcare system features good accessibility, comprehensive coverage, short waiting times, and relatively low cost [31]. Almost all medical treatments are covered under the NHI program. Moreover, all patients with cancer can apply for a catastrophic illness certificate. Patients with this certification can access medical care without copayments [32]. As a result, cost is not a deterrent to accepting more aggressive treatment as neither the patient nor the family has to pay any additional fees to receive it.

The results of this study also suggest that patients with both cancer and dementia tend to undergo fewer advanced diagnostic procedures, a factor not examined in other studies. Our finding may be explained by the fact that unlike emergent invasive interventions, such as endotracheal intubation or CPR, doctors order diagnostic studies at their own discretion and discussion with family members. Therefore, if a patient with dementia is near the end of life, the doctor may decide there is no benefit to the patient in pursuing further diagnostic procedures and will not even suggest it. Family members tend to accept this decision without seeing it as having an impact on their view of filial piety. Finally, practical difficulties can also play a role in decisions pertaining to advanced testing. For example, dementia or behavioral and spatial temporal disorders can make performing procedures such as MRIs, scans, or panendoscopy more difficult, changing the risk–benefit and cost–benefit ratios in such cases [6].

The primary strength of the current study is its nationwide population-based design. The large-scale database we used provided a sufficient sample size to allow rigorous research to be conducted. However, this study also suffers from some limitations. First, the prevalence of dementia was lower than that reported in the literature [33]. The low prevalence that we observed may be related to the stricter screening criteria we adopted. To be included in this research, patients had to possess a catastrophic illness certification for dementia or have been diagnosed with dementia by a neurologist or a psychiatrist. Patients who were diagnosed with dementia by other specialists were not included in this study. However, we believe that using stricter inclusion criteria ensured the accuracy of the dementia diagnoses. Second, the severity of dementia may affect the decision making. However, the claims data does not report the severity of dementia and the patients’ level of functioning. Therefore, we do not know if our results are generalizable to all patients with dementia, and further studies may be necessary to explore this issue. Third, evidence obtained in cohort studies can be biased by unmeasured or unknown confounders. Although we attempted to control for some potential confounding factors by including suitable matching and covariates adjustment, other confounders may still exist.

In summary, this study revealed that patients with both cancer and dementia were more likely to be cared for in the ICU and to receive invasive procedures, but less likely to receive chemotherapy, palliative care, or advanced diagnostic testing than those without dementia. Further investigation to gain a better understanding of the decision-making process for patients with dementia and cancer is required.

## MATERIALS AND METHODS

### Data sources

In 1995, Taiwan instituted the NHI program, which is a single-payer program administered by the government. NHI covers approximately 99% of the Taiwanese population and includes contracts with 97% of the hospitals and clinics in Taiwan. We used the NHI Research Database containing NHI claims data from 2000 to 2012 obtained from the National Health Research Institute (NHRI). This data includes registries and claims from contracted health-care facilities. After receiving NHRI approval, we obtained medical records from the Catastrophic Illness Patient Database and the registry of beneficiaries, as well as ambulatory claims and inpatient care claims. Concerning data security and the patient's privacy, personal identification was encrypted before releasing it from the database. Patient diagnoses were determined using the International Classification of Disease, 9th Revision, Clinical Modification (ICD-9-CM). This study was approved by the Institutional Review Board of the Tzu Chi Medical Center.

### Study population

We used the Catastrophic Illness Patient Database to identify a study cohort that included patients who had been newly diagnosed with one of the six most common cancers in Taiwan between 2000 and 2012. Specifically, these were colorectal (ICD-9 codes 153–154), liver (ICD-9 code 155), lung (ICD-9 code 162), breast (ICD-9 code 174), oral (ICD-9 codes 140–141, 143–146, 148–149), and prostate cancers (ICD-9 code 185). Patients were excluded from the study if they 1) had more than one cancer diagnosis, 2) were younger than 20 years old, 3) remained alive during the follow-up period, 4) had history of traffic accident before death, or 5) did not receive inpatient care within the final month prior death.

To specify a dementia group, we identified patients with cancer from the study cohort who had also been assigned an ICD-9 diagnostic code for dementia during the study period (i.e., ICD-9 codes 290.0-290.4 and 331.0). The ICD-9 dementia codes had to be 1) shown on a catastrophic illness certification for dementia, 2) assigned by a neurologist or a psychiatrist during a hospital stay, or 3) assigned by a neurologist or a psychiatrist in an outpatient clinic at least twice in the same year.

We then matched four patients with cancer but without a dementia diagnosis with each patient with cancer who had a dementia diagnosis. Patients were matched by age, gender, type of primary cancer, year of death, and length of time between cancer diagnosis and death (Figure 1). To account for the possible impact of disease progression on decisions pertaining to end-of-life care, we analyzed patients matched for the length of time between cancer diagnosis and death, comparing variables among those dying within 1 month of diagnosis and also among those dying within 3 months of diagnosis.

### Research variables

We used medical records to identify the comorbid conditions each patient had during the year prior to death, allowing calculation of the Charlson comorbidity index [34]. Demographic and clinical characteristics included age, gender, type of primary cancer, year of death, and length of time between cancer diagnosis and death. We also evaluated the socioeconomic status of patients using income-related insurance premiums as a proxy for income. Specifically, insurance premium was classified into four categories: ≥25,001 New Taiwan dollars (NTD), 15,841–25,000 NTD, 1–15,840 NTD, or financially dependent (such as the unemployed, students, children, and elderly persons with no salary).

### Study outcomes

In this study, we investigated how dementia influenced the medical care, such as aggressive treatments or invasive procedures, which a patient with cancer received during the final 1 and 3 months of their lives. More specifically, for patients with and without dementia, we compared 1) length of hospital stay, intensive care unit (ICU) stay, and hospice care; 2) anticancer chemotherapy; 3) invasive procedures, including CPR, endotracheal intubation, mechanical ventilation, urinary catheterization, and feeding tubes; and 4) advanced diagnostic tests, including CT or MRI or sonography, panendoscopy, colonoscopy, bone scans, and positron emission tomography (PET) scans.

### Statistical analysis

Continuous variables were analyzed using independent Student's t-tests and a multiple logistic regression model was used to determine whether a dementia diagnosis influenced the likelihood of utilization of types of care, invasive procedures, and advanced diagnostic testing. Corrections were made to account for age, gender, type of primary cancer, Charlson comorbidity index, year of death, length of time between cancer diagnosis and death, and insurance premiums (Table 1). Results are reported as adjusted odd ratios (ORs) with 95% confidence intervals (CIs). A value of p < 0.05 is considered statistically significant. All statistical analyses were performed using SAS 9.4 (SAS Institute, Inc., Cary, NC).

## Abbreviations

OR: odds ratio
CI: confidence interval
ICU: intensive care unit
CPR: cardiopulmonary resuscitation
CT: computed tomography
MRI: magnetic resonance imaging
PET: positron emission tomography
NHI: National Health Insurance
NHRI: National Health Research Institute
NTD: New Taiwan dollars
